# Bruton's Tyrosine Kinase and Protein Kinase C µ Are Required for TLR7/9-Induced IKKα and IRF-1 Activation and Interferon-β Production in Conventional Dendritic Cells

**DOI:** 10.1371/journal.pone.0105420

**Published:** 2014-08-29

**Authors:** Yan-Feng Li, Koon-Guan Lee, Xijun Ou, Kong-Peng Lam

**Affiliations:** 1 Immunology Group, Bioprocessing Technology Institute, Agency for Science, Technology and Research, Singapore, Singapore; 2 Department of Physiology, Yong Loo Lin School of Medicine, National University of Singapore, Singapore, Singapore; 3 Department of Microbiology, Yong Loo Lin School of Medicine, National University of Singapore, Singapore, Singapore; 4 Department of Pediatrics, Yong Loo Lin School of Medicine, National University of Singapore, Singapore, Singapore; University of Nebraska – Lincoln, United States of America

## Abstract

Stimulation of TLR7/9 by their respective ligands leads to the activation of IκB kinase α (IKKα) and Interferon Regulatory Factor 1 (IRF-1) and results in interferon (IFN)-β production in conventional dendritic cells (cDC). However, which other signaling molecules are involved in IKKα and IRF-1 activation during TLR7/9 signaling pathway are not known. We and others have shown that Bruton's Tyrosine Kinase (BTK) played a part in TLR9-mediated cytokine production in B cells and macrophages. However, it is unclear if BTK participates in TLR7/9-induced IFN-β production in cDC. In this study, we show that BTK is required for IFN-β synthesis in cDC upon TLR7/9 stimulation and that stimulated BTK-deficient cDC are defective in the induction of IKKα/β phosphorylation and IRF-1 activation. In addition, we demonstrate that Protein Kinase C µ (PKCµ) is also required for TLR7/9-induced IRF-1 activation and IFN-β upregulation in cDC and acts downstream of BTK. Taken together, we have uncovered two new molecules, BTK and PKCµ, that are involved in TLR7/9-triggered IFN-β production in cDC.

## Introduction

Conventional dendritic cells (cDC) and plasmacytoid dendritic cells (pDC) recognize single-stranded (ss) RNA and unmethylated CpG DNA via Toll-like receptors (TLR) 7 and 9, respectively, to initiate innate immune responses against viruses and bacteria [1], [2], [3], [4]. TLR7 and TLR9 are endosome-bound, leucine-rich-repeat containing type I transmembrane glycoproteins and they signal via the adaptor MyD88 to induce the production of proinflammatory cytokines and type I interferons (IFN) [2], [5]. The secretion of proinflammatory cytokines is dependent on the activation of the transcription factor nuclear factor κB (NF-κB) while the synthesis of type I IFN is dependent on the combined activation of NF-κB and IFN regulatory factors (IRF) [6], [7]. The IRF family of transcription factors comprises nine members (IRF-1–9), and which IRF is activated depends on the specific cell type involved. For instance, pDC rapidly produce high amounts of IFN-α through the induction of the transcription factor IRF-7 [8], [9], [10], whereas IRF-1 is activated and translocated to the nucleus to induce IFN-β systhesis in cDC [11], [12].

IκB kinase α (IKKα) has been shown to associate and activate IRF-1 during TLR7/9 signaling in cDC [12] and acts specifically via IRFs to induce the production of IFN. Given the importance of TLRs in host defense against pathogens, dissection of TLR7/9 signaling pathways becomes an important research focus. However, the TLR7/9 signaling pathway leading to IKKα and IRF-1 activation in cDC has been relatively unexplored and it is currently not known which other signaling molecules participate in TLR7/9-signaling leading to IRF-1 activation and IFN-β synthesis. Thus in this current study, we set out to determine the upstream signaling molecules that might play a role in TLR7/9-induced activation of IKKα and IRF-1 leading to IFN-β production.

Bruton's tyrosine kinase (BTK) is a member of the Tec family of protein tyrosine kinases and has previously been shown to play important roles in B cell development, activation and survival. Mutations in BTK are known to lead to X-linked agammaglobulinemia (*XLA*) in humans and X-linked immunodeficiency (*xid*) in mice [13]. BTK has been shown to be activated by B cell antigen receptor (BCR) [14], cytokine receptors [15], [16], as well as TLR [17], [18], [19], [20] engagement. It is well documented that TLR-induced NF-κB activation is defective in BTK-deficient cells [21], [22], [23]. We and others also demonstrated that BTK played a critical role in MyD88-dependent TLR-signaling of proinflammatory cytokine production in B cells [17], macrophages [18], [20] and cDC [24], [25]. In addition, we recently showed that BTK is required to phosphorylate TLR3 for IFN-β production [26]. However, it is unclear if BTK plays a role in TLR7/9-induction of IFN-β synthesis in cDC.

In addition to BTK, it is conceivable that other signaling molecules could contribute to TLR7/9-induced IFN-β production. Various PKCs have been found to be important for TLR-induced production of proinflammatory cytokines [27], [28] and PKCα has been shown to be involved in TLR3-induced IFN-β synthesis [29]. Protein Kinase C (PKC) is a class of serine/threonine kinase that is subdivided into three main groups, namely conventional PKCs, novel PKCs, and atypical PKC [30], [31]. Protein kinases D, consisting of PKD1 (also known as PKCµ), PKD2, and PKD3, are extended family members of PKC [32]. It is also not clear currently whether PKC participates, and if so, which PKC member is involved in TLR7/9-induced IFN-β production in cDC. In this paper, we undertook to examine if both BTK and PKC are involved in TLR-7/9 activation of IFN-β production in cDC.

## Materials and Methods

### Ethics statement

All experiments and procedures were performed in compliance with Guidelines on the Care and Use of Animals for Scientific Purposes of the National Advisory Committee for Laboratory Animal Research (NACLAR) of Singapore. The protocol was approved by the Institutional Animal Care and Use Committee of the Biological Resource Center (BRC) of Agency for Science, Technology and Research (A*STAR) (Authorized IACUC No 110611). Steps were taken to minimize animal suffering.

### Mice

Wild type C57BL/6 mice were obtained from the BRC, and *btk^−/−^* mice were obtained from The Jackson Laboratory.

### Cells and transfection

Conventional dendritic cells (cDC) were differentiated as described [33], [34]. Briefly, bone marrow (BM) cells were cultured in Dulbecco's modified Eagle's medium (DMEM) supplemented with 100 units/ml penicillin, 100 µg/ml streptomycin, 2 mm l-glutamine, 50 µm 2-mercaptoethanol, 10% heat-inactivated fetal calf serum, and 1% supernatant of granulocyte-macrophage colony-stimulating factor-transduced X-63 cells. After 6 to 7 days of culture, cDC were purified using anti-CD11c monoclonal antibody-coupled magnetic beads (Miltenyl Biotech). For PKCµ knock down studies, 5 µg of PKCµ siRNA or scrambled siRNA (Santa Cruz) were transfected into purified cDC (1×10^6^ cells per reaction) using Amaxa Nucleofector device and reagents as per manufacturer's instructions (Amaxa). Transfected cDC were maintained for an additional 24 h before stimulation.

### Reagents

The following reagents were purchased and used in the study: TLR7 agonist R848 and TLR9 ligand CpG-ODN 1668 (CpG) (Invivogen); PKC inhibitors Gö 6976 and non-inhibitory analog Gö 6983 (Calbiochem). Antibodies used for immunoblot analyses were from Santa Cruz: anti-BTK, anti-IKKα/β, anti-PKCµ, anti-IRF-1, anti-HDAC-1, anti-β-actin, donkey anti-goat IgG-horseradish peroxidase, goat anti-rabbit IgG-horseradish peroxidase, and goat anti-mouse IgG-horseradish peroxidase; from Cell Signaling: anti-phospho-BTK (Tyr^223^), anti-phospho-PKCµ (Ser^916^) and anti-phospho-IKKα (Ser^180^)/IKKβ (Ser^181^). The anti-phosphotyrosine horseradish peroxidase-conjugated antibody (4G10) was from Upstate Biotech.

### Immunoprecipitations and western blot analyses

Cells were treated for various times as shown with the indicated stimuli, followed by immunoprecipitations and western blot analyses as described previously [17]. 10 million wild type and *btk^−/−^* cDC were plated onto 3cm dish, left untreated or stimulated with R848 or CpG and then washed with cold PBS and lysed on ice for 30 mins in 500 µl phospholysis buffer containing 1% Nonidet P-40, 10 mM Tris-HCl, pH 8.0, 150 mM NaCl, 1 mM EDTA, 0.2 mM Na_3_VO_4_, and a cocktail of protease inhibitors (Roche). Whole cell lysates were obtained after centrifugation at 13,000 rpm for 10 mins at 4°C. For immunoprecipitation studies, cell lysates were pre-cleared with Protein A/G Plus-agarose in the presence of goat IgG (Santa Cruz) for 1 h at 4°C. 3 µg of anti-BTK antibody and 20 µl beads were added to the pre-cleared lysate and rotated for 3 h at 4°C. Subsequently, beads were washed 3 times with phospholysis buffer and boiled in loading buffer for 10 mins. For Western blot analyses, whole cell lysates or immunoprecipitated proteins were electrophoresed in 10% SDS-polyacrylamide gels and transferred onto immunoblot polyvinylidene difluoride membranes (Millipore). The membranes were then probed with antibodies of interest (1∶1000) and normalized for total protein or β-actin. To examine nuclear translocation of IRF-1, nuclear proteins from untreated or stimulated wild type and *btk^−/−^* cDC were prepared using NE-PER Nuclear and Cytoplasmic Extraction Reagents according to manufacturer's instruction (Pierce). Nuclear proteins were probed with anti-IRF-1 and normalized with anti-HDAC-1 antibodies.

### Measurement of IFN-β production by ELISA or Quantitative RT-PCR

Cells were treated for 6 h with the indicated stimuli, and IFN-β production was measured by ELISA as described [12]. Briefly, untreated or CpG-stimulated wild type and *btk^−/−^* cDC cells were cultured in 24-well plates at 1×10^6^ cells/ml. At 6 h poststimulation, supernatants were harvested and stored at -20°C or immediately assayed for cytokine production. The concentrations of IFN-β were determined using commercial ELISA kit (PBL Interferon Source) according to manufacturer's instructions. For quantification of IFN-β mRNA synthesis, total RNA was extracted from wild type and *btk^−/−^* cDC at 2 h post-stimulation using Trizol (Invitrogen), and cDNA was synthesized with Superscript II reverse transcriptase as per manufacturer's protocol (Invitrogen). Quantitative PCR was performed on an Applied Biosystems 7500 real time PCR system using the following primers: IFN-β, 5'-CAGCTCCAAGAAAGGACGAAC-3' and 5'-GGCAGTGTAACTCTTCTGCAT-3'; β-actin, 5′-AGATGACCCAGATCATGTTTGAGA-3′ and 5′-CACAGCCTGGATGGCTACGTA-3′.

### Immunofluorescence

0.1 million WT or *btk^−/−^* cDC were plated on glass-bottomed 35 mm dish, stimulated for 1 h with R848 (1 µM) in the presence or absence of Gö 6976 or Gö 6983 (30 min pre-treatment, 37°C) and then fixed in 4% paraformaldehyde (5 min, RT), washed in PBS, permeabilized with 1% Triton X-100 in PBS for 10 minutes at RT and rinse well with PBS. The dish was then blocked with 3% BSA (1 h, RT). Cells were later incubated for 16 hrs at 4°C with anti-IRF-1 antibody (1∶50) in PBS containing 3% BSA. After incubation with secondary anti-Rabbit IgG PE antibody (1∶50, 1 h, RT), cells were washed and mounted with coverslips using VECTASHIELD Mounting Medium with DAPI (Vector Laboratories). Images of cells were taken and analyzed using an Olympus Fluoview Version 2.1 software using 100X objectives with oil.

### Statistics

Statistical analysis was performed using Student's *t* test (Prism). *p* values of <0.05 were considered significant.

## Results

### BTK is phosphorylated upon TLR7/9 engagement in cDC

**Figure 1:**
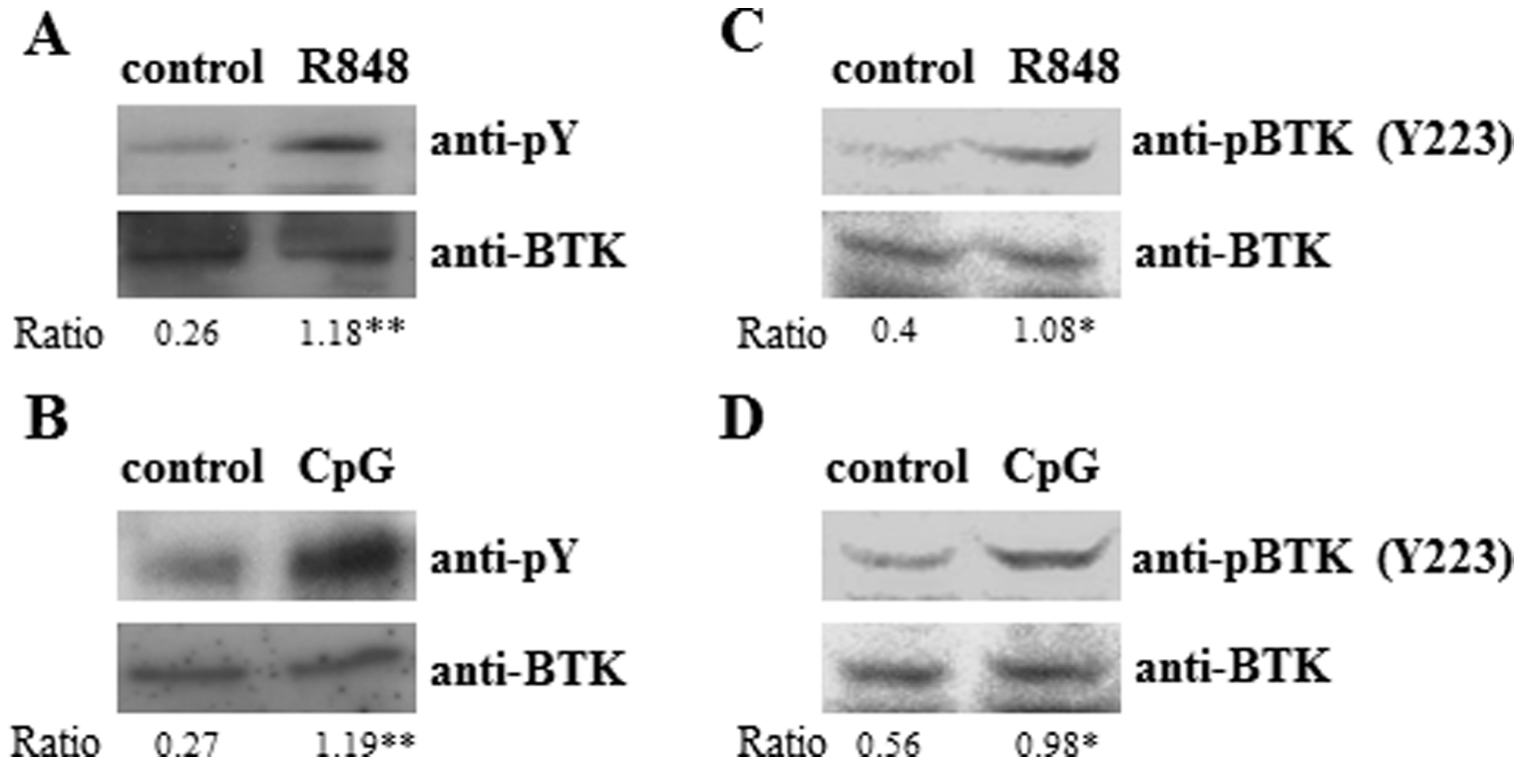
Bruton's tyrosine kinase is phosphorylated upon TLR7/9 stimulation in cDC. Purified wild type cDC (10 million cells per 3 cm dish) were untreated or stimulated for 5 mins with either (A) TLR7 agonist (R848, 1 µM) or (B) TLR9 ligand (CpG ODN-1668, 0.1 µM). Cells were then lysed in 500 µl lysis buffer for 30 min on ice and BTK was immunoprecipitated and probed with anti-phospho-tyrosine antibody (pY, 4G10) and reprobed with anti-BTK antibodies. Total lysates after R848 (C) or CpG (D) treatment were also immunoblotted with anti-phospho-BTK (Y223) and re-probed with BTK antibodies for equal loading of BTK. Results shown are representative of three independent experiments. The intensities of the bands were quantified by ImageJ and the ratio of 4G10 or phopspho-BTK (Y223) over BTK in each sample was then calculated and presented. *p<0.05, **p<0.005 (Student's *t* test), compared to untreated cells.

To determine if BTK is involved in TLR7/9 signaling, we differentiated cDC from bone marrow (BM) precursors and stimulated them with the TLR7 agonist R848 or the TLR9 ligand CpG ODN-1668 (CpG) and examined if BTK is phosphorylated upon TLR engagement. After treatment of wild type cDC with R848 or CpG, total BTK was immunoprecipitated and probed for tyrosine phosphorylation using the phospho-tyrosine specific antibody 4G10. As shown in Fig. 1A, BTK was activated, as indicated by its tyrosine phosphorylation status upon stimulation of cDC with R848. Similarly, stimulation of cDC with CpG also led to the phosphorylation of BTK (Fig. 1B). To confirm the phosphorylation of BTK, the same cell lysates were also subjected to immunoblot with anti-phospho-BTK (Y223) antibody. Consistently we observed an increase in the amount of phosphorylated BTK in cDC after treatment of these cells with either R848 (Fig. 1C) or CpG (Fig. 1D). Thus, these data indicated that BTK was activated during TLR7/9 signaling in cDC. This result adds onto our previous demonstration of a role for BTK in TLR9 signaling in B cells [17] and other cell types [18], [20], [35], [36]. In addition, our current finding also demonstrates that BTK is activated upon TLR7 stimulation in cDC, which has not been previously documented.

### BTK is involved in IFN-β production following TLR7/9-stimulation in cDC

**Figure 2:**
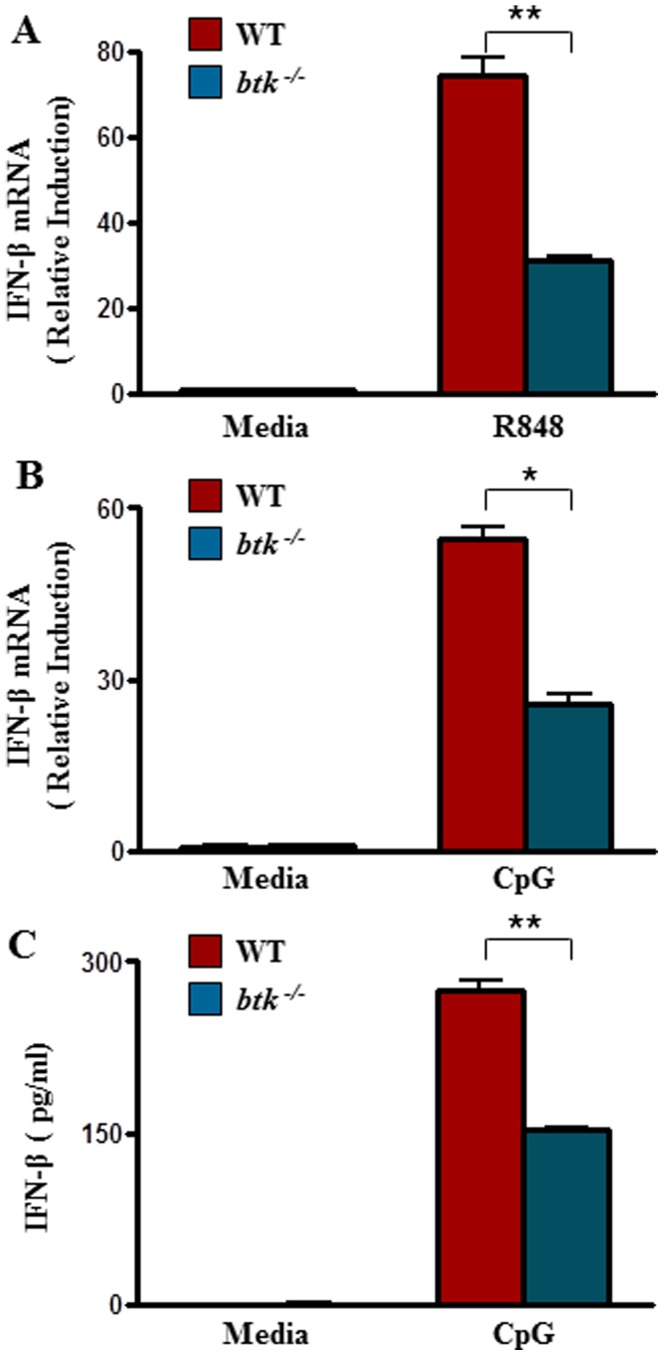
TLR7/9-induced IFN-β production is defective in *btk^−/−^* cDC. Purified wild type or *btk^−/−^* cDC were untreated or stimulated for 2 h with either (A) R848 (1 µM) or (B) CpG (0.1 µM). IFN-β mRNA was quantified by qRT-PCR with primers as listed in materials and methods. IFN-β mRNA expression was normalized to β-actin, and the value from un-stimulated cells was set as 1. Numbers represent mean±SEM of three experiments. **, p < 0.005; *, p < 0.05 (Student's *t* test). (C) IFN-β protein level in the supernatants of untreated (media) or CpG (0.1 µM, 6 h) stimulated cDC were also measured via ELISA using known standards. *, p < 0.05 (Student's *t* test). Error bars show the standard error calculated from three biological replicates.

Signaling through TLR7/9 leads to the secretion of IFN-α in plasmacytoid dendritic cell (pDC) [3], [10] and IFN-β in cDC [12]. Previously our laboratory has demonstrated that BTK played a critical role in TLR9-induced production of proinflammatory cytokines such as IL-6, IL-12 and TNF-α [17] in B cells. Here, we asked if BTK participates in TLR7/9-induced production of IFN-β in cDC. As shown in Fig. 2, the synthesis of IFN-β mRNA was highly induced in wild type cDC when they were stimulated for 2 h with R848 (A) or CpG (B). By contrast, there was a significant reduction of IFN-β mRNA induction in R848 or CpG-stimulated *btk* ^−/−^ cDC. This finding was further confirmed when we measured secreted IFN-β protein level via ELISA from CpG stimulated *btk* ^−/−^ cDC (Fig. 2C). Hence, the data indicated that BTK is important for TLR7/9-induced IFN-β production in cDC.

### Activation of IκB kinase α and IRF-1 is impaired in TLR7/9-stimulated *btk* ^−/−^ cDC

So far, not much is known of the TLR7/9 signaling pathway that leads to IFN-β production in cDC. The only well-established molecules in the activation process are IKKα that induces IRF-1 in cDC [12] and IRF-7 in pDC [9], [10]. BTK had previously been reported to be required for IκB kinase activation in B lymphocytes [21] and IRF-3 induction in macrophages [26]. However, it is not known whether BTK plays a role in IKKα and IRF-1 activation in cDC.

**Figure 3:**
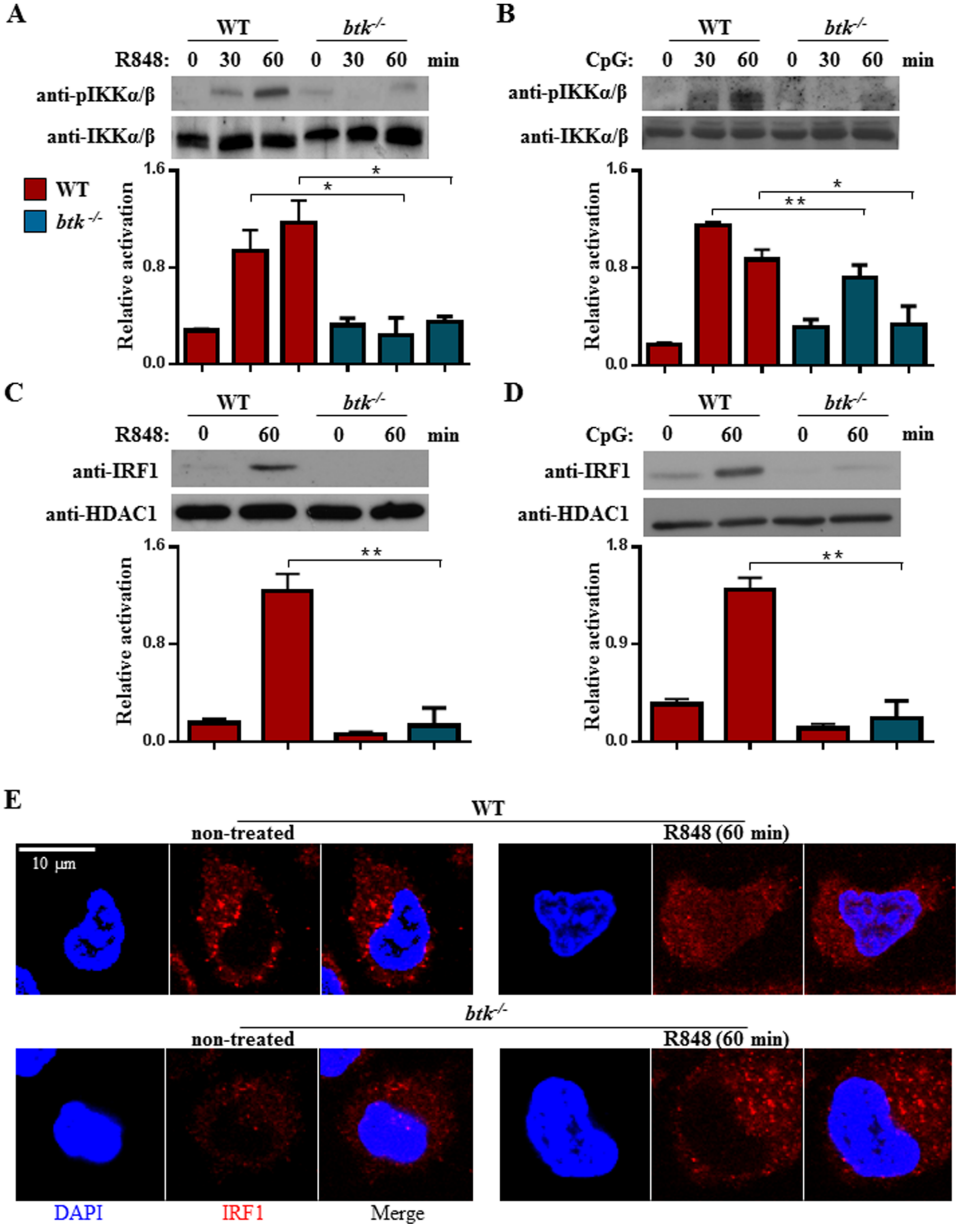
Impaired activation of IKKα/β and IRF-1 in TLR7/9-stimulated *btk^−/−^* cDC. Wild type and *btk^−/−^* cDC were stimulated with 1 µM R848 (A) or 0.1 µM CpG (B) for various times as indicated and IKKα/β activation was examined via immunoblot analyses using anti-phospho-IKKα (Ser^180^)/IKKβ (Ser^181^) antibody. The anti-IKKα/β blot was included as loading control. Densitometric ratios of phospho-IKKα (Ser^180^)/IKKβ (Ser^181^) over IKKα/β are graphed and shown in the lower panels of A and B. *p<0.05, **p<0.005 (Student's *t* test). (C & D) Nuclear translocation of IRF-1 in cDC that were stimulated for 1 h with R848- (C) or CpG- (D). Nuclear extracts were obtained from non-treated or 1 h stimulated wild type and *btk^−/−^* cDC and examined for the presence of IRF-1 via western blot analyses. The anti-HDAC1 blot was included as loading control. The ratio of densitometric values of IRF-1 over HDAC1 is shown in lower panels of C and D. Numbers represent mean±SEM of three experiments. **p<0.005 (Student's *t* test). (E) Immunofluorescence confocal studies of IRF-1 localization in wild type and *btk^−/−^* cDC that were untreated or stimulated for 1 h with 1 µM R848. Bar = 10 µM. Results shown are representative of at least three independent experiments.

To investigate whether BTK is involved in the TLR7/9 signaling pathway leading to IKKα induction in cDC, we examined the activation of IKKα/β using an anti-phospho- IKKα/β specific antibody. As shown in Fig. 3A, TLR7-induced activation of IKKα/β was markedly attenuated in *btk^−/−^* cDC as compared with wild type controls when these cells were treated with R848 agonists. The reduced phosphorylation pattern of IKKα/β was observed across the various time points examined. Similar defects were also observed when *btk^−/−^* cDC were stimulated with the TLR9-ligand, CpG (Fig. 3B). Hence, BTK is critical for the optimal activation of IKKα/β during TLR7/9 engagement.

Induction of IKKα in TLR7/9-activated cDC is known to lead to the phosphorylation and nuclear translocation of IRF transcription factors that drive the synthesis of type I IFNs [10], [12]. IFN-β gene expression is regulated by different IRF family members in different cell types and upon different stimuli [9], [37]. cDC utilizes IRF-1 for TLR9-induced IFN-β production [11], [12], [38]. To determine if the activation of IRF-1 was affected in TLR7/9-stimulated *btk^−/−^* cDC, we examined the nuclear translocation of IRF-1 in these cells. As shown in Fig. 3C, the engagement of TLR7 by R848 led to the activation and nuclear translocation of IRF-1 in wild type cDC. By contrast, the activation of IRF-1 was largely compromised in R848-treated *btk^−/−^* cDC. Similar finding was also observed for CpG-stimulated *btk^−/−^* cDC (Fig. 3D). Furthermore, when confocal imaging microscopy was used to analyze IRF-1 localization, we observed increased translocation of IRF-1 into the nucleus of cells after R848 stimulation. By contrast, IRF-1 translocation to the nucleus was not evident in *btk^−/−^* cDC (Fig. 3E). Collectively, our results indicated that the signal transduced by BTK is required to activate IKKα/β and subsequently, IRF-1 in TLR7/9-stimulated cDC.

### PKCµ phosphorylation is impaired in TLR7/9-stimulated *btk* ^−/−^ cDC

BTK is known to interact with a number of signaling molecules in various immune and cytokine receptor signaling [39]. One of these molecules is Protein Kinase C (PKC) [40]. Various PKC isoforms are known to be involved in the signaling cascade leading to NF-κB activation [41], [42], [43], [44]. For example, PKCβ is critical for B cell-receptor induced IKK activation [43]. On the other hand, PKCα has been shown to be important for MyD88-dependent TLR signaling leading to proinflammatory cytokine production in DC [27] and for TLR3-induced IRF-3 activation and IFN-β synthesis [29]. PKCµ (PKD1) has also been shown to be important for TLR9 [45] and other MyD88-dependent TLR signaling of pro-inflammatory cytokine production in cDC [46]. However, in all these studies, it is not clear if PKCµ is activated during TLR7/9 signaling and if it played a role in TLR7/9-induced IFN-β production and if BTK has a role in the activation of PKCµ.

**Figure 4:**
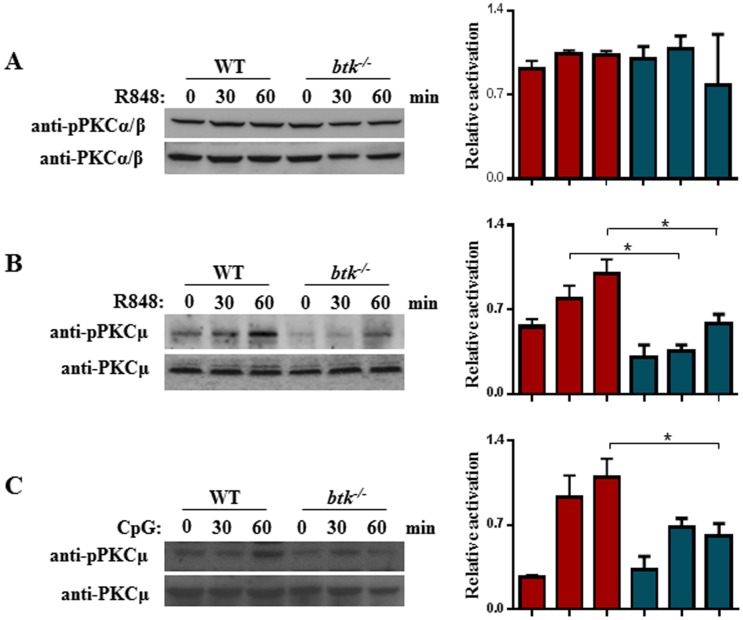
Defective PKCµ activation in TLR7/9-stimulated *btk^−/−^* cDC. Wild type and *btk^−/−^* cDC were stimulated with R848 (A & B, 1 µM) or CpG (C, 0.1 µM) for various times as indicated and the activation of PKCα/β (A) or PKCµ (B & C) was examined by immunoblotting with anti-phospho-PKCα/β (Thr^638/641^) or anti-phospho-PKCµ (Ser^916^) antibodies. Immunoblot membranes were re-probed with anti-PKCα/β or anti-PKCµ antibodies to check for equal loading of lysates. The ratios of the intensity of phospho-PKCα/β (Thr^638/641^) over PKCα/β (A) and phospho-PKCµ (Ser^916^) over PKCµ (B & C) are shown on the right. Numbers represent mean±SEM of three experiments. *p<0.05 (Student's *t* test). Gels shown are representative of three independent experiments.

First, to determine if any PKC would be involved and acts downstream of BTK in TLR7/9-induced activation of IFN-β production, we examined if the activation of any of the PKC isoforms would be compromised in R848 or CpG-stimulated *btk^−/−^* cDC. Surprisingly, PKCα/β phosphorylation was found to be comparable between TLR7-stimulated wild type and *btk^−/−^* cDC (Fig. 4A). However as shown in Fig. 4B & C, the phosphorylation of PKCµ was up-regulated in WT cDC treated with R848 or CpG. By contrast, the activation of PKCµ was severely attenuated across all time points tested in R848-stimulated *btk^−/−^* cDC compared with similarly-treated wild type controls. The activation of PKCµ was also found to be defective in TLR9-stimulated *btk^−/−^* cDC (Fig. 4C). Thus, PKCµ was activated by TLR7/9 engagement in cDC and the absence of BTK in cDC specifically impaired the activation of PKCµ. These data suggest that PKCµ is involved and acts downstream of BTK in TLR7/9-signaling in cDC.

### Reduced expression of PKCµ impairs TLR7/9-induced IFN-β synthesis

**Figure 5:**
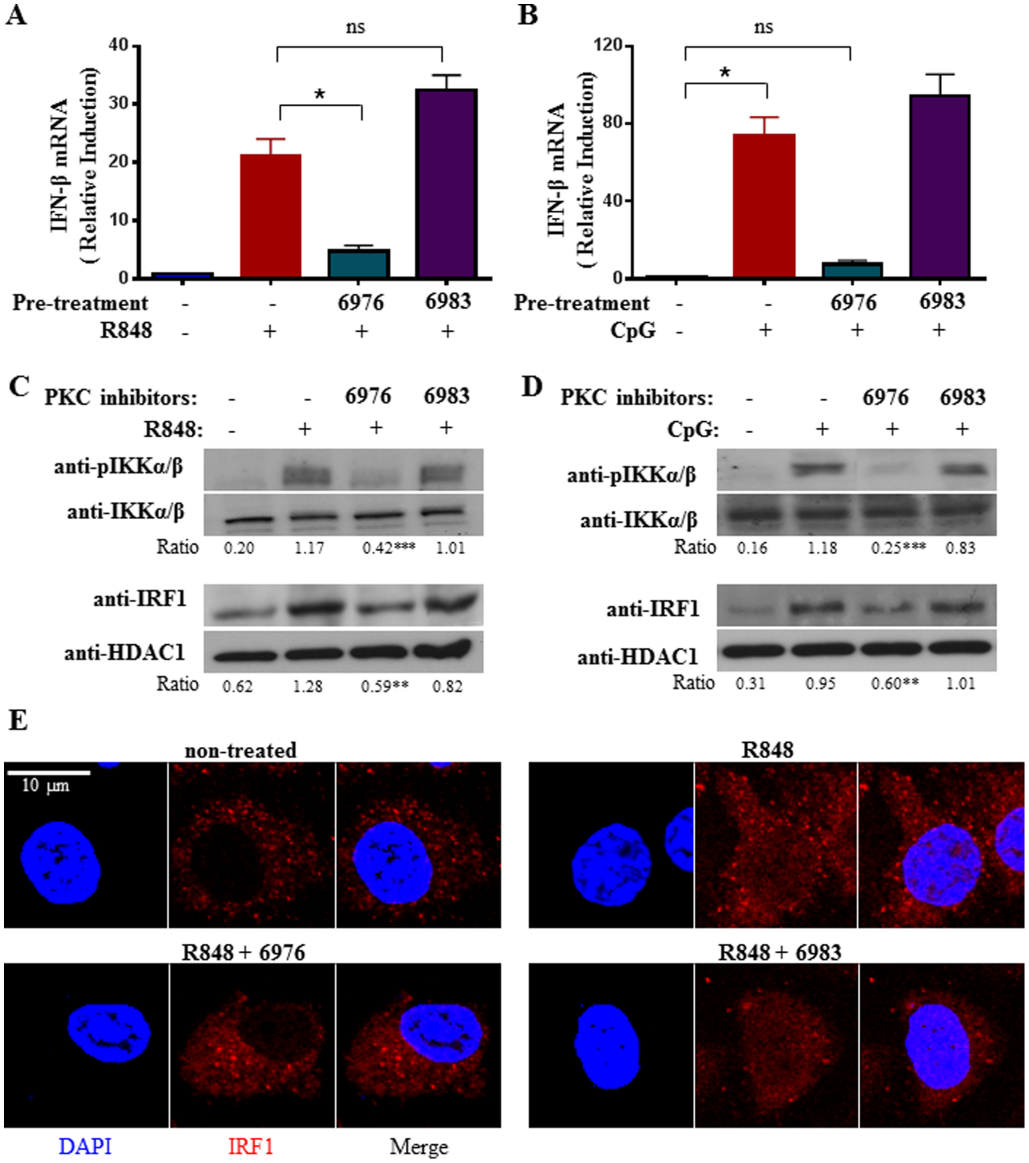
Chemical inhibition of PKCµ activity compromises TLR7/9-inducd IKKα/β and IRF-1 activation and IFN-β synthesis in cDC. Wild type cDC were non-treated or pretreated for 30 mins with PKC inhibitors Gö 6976 (0.1 µM) or the non-inhibitory analog Gö 6983 (1 µM), followed by stimulation with R848 (1 µM) (A) or CpG (0.1 µM) (B) for 2 h and IFN-β mRNA expression was measured. Data were analyzed as in Fig. 2A. (C & D), Western blot analyses of IKKα/β and IRF-1 activation in PKCµ-inhibited TLR7/9-stimulated cDC. Wild type cDC were non-treated or pretreated for 30 mins with PKC inhibitors Gö 6976 (0.1 µM) or the non-inhibitory analog Gö 6983 (1 µM), followed by stimulation with R848 (1 µM) (C) or CpG (0.1 µM) (D) for 1 h. Activation of IKKα/β was examined using anti-phospho-IKKα (Ser^180^)/IKKβ (Ser^181^) antibody. The anti-IKKα/β blot served as loading control. Determination of IRF-1 activation was performed using anti-IRF-1 antibodies after nuclear extraction. The anti-HDAC1 immunoblots served as loading control. The ratios of the intensities of phospho-IKKα (Ser^180^)/IKKβ (Ser^181^) over IKKα/β and IRF-1 over HDAC1 are shown below the gels. **p<0.005, ***p<0.001 (Student's *t* test), compared to R848- or CpG-treated cells without PKCµ inhibition. (E) Confocal immunofluorescence study of IRF-1 localization in PKCµ-inhibited and R848-stimulated cDC. Wild type cDC were untreated or pre-treated for 30 mins with Gö 6976 (0.1 µM) or 6983 (1 µM), followed by stimulation with 1 µM R848 for 1 h. Bar  = 10 µM. Results shown are representative of at least three independent experiments.

Our data above suggested that PKCµ is activated by TLR7/9 stimulation and its activation is impaired in the absence of BTK. Although PKCµ has been implicated in TLR9 signaling of proinflammatory cytokine production [45], it has not been shown whether PKCµ is involved in TLR7/9-induced IFN-β production. To address this, we inhibit PKCµ activity using a specific chemical inhibitor Gö 6976 [47]. We pre-treated cDC with this inhibitor and subsequently stimulated the cells with either R848 or CpG and examined their induction of IFN-β mRNA. As shown in Fig. 5A & B, Gö 6976-treated and hence, PKCµ-inhibited cDC failed to induce IFN-β mRNA synthesis upon TLR7/9 stimulation. By contrast, non-treated or cDC treated with Gö 6983, a non-inhibiting analog of Gö 6976, were able to robustly synthesize IFN-β when they were stimulated with R848 or CpG. These data suggest that PKCµ played a role in TLR7/9-induced IFN-β production.

To determine if PKCµ acts in the same pathway as IKKα and IRF-1 in inducing IFN-β production, we examined the activation of IKKα/β and IRF-1 in R848 or CpG-stimulated cDC with or without Gö 6976 or Gö 6983 pre-treatment. As shown in Fig. 5C, stimulation of cDC with R848 induces IKKα/β and IRF-1 activation, as assessed by the phosphorylation of IKKα/β and nuclear localization of IRF-1. However, the extent of IKKα/β phosphorylation and IRF-1 nuclear localization were very much reduced in Gö 6976-treated and R848-stimulated cells. By contrast, the activation of IKKα and IRF-1 remain intact in Gö 6983-treated R848-stimulated cells. The same observations were also found to hold true for Gö 6976 or Gö 6983 pre-treated cDC that were also stimulated with GpG (Fig. 5D). Furthermore, when confocal imaging microscopy was used to check the cellular localization of IRF-1, we observed increased IRF-1 in the nucleus after R848 stimulation. However, nuclear IRF-1 was very much reduced in Gö 6976 pre-treated cDC (Fig. 5E). Thus, inhibition of PKCµ activity perturbs IKKα/β and IRF-1 activation and impairs IFN-β synthesis in TLR7/9-stimulated cDC.

**Figure 6:**
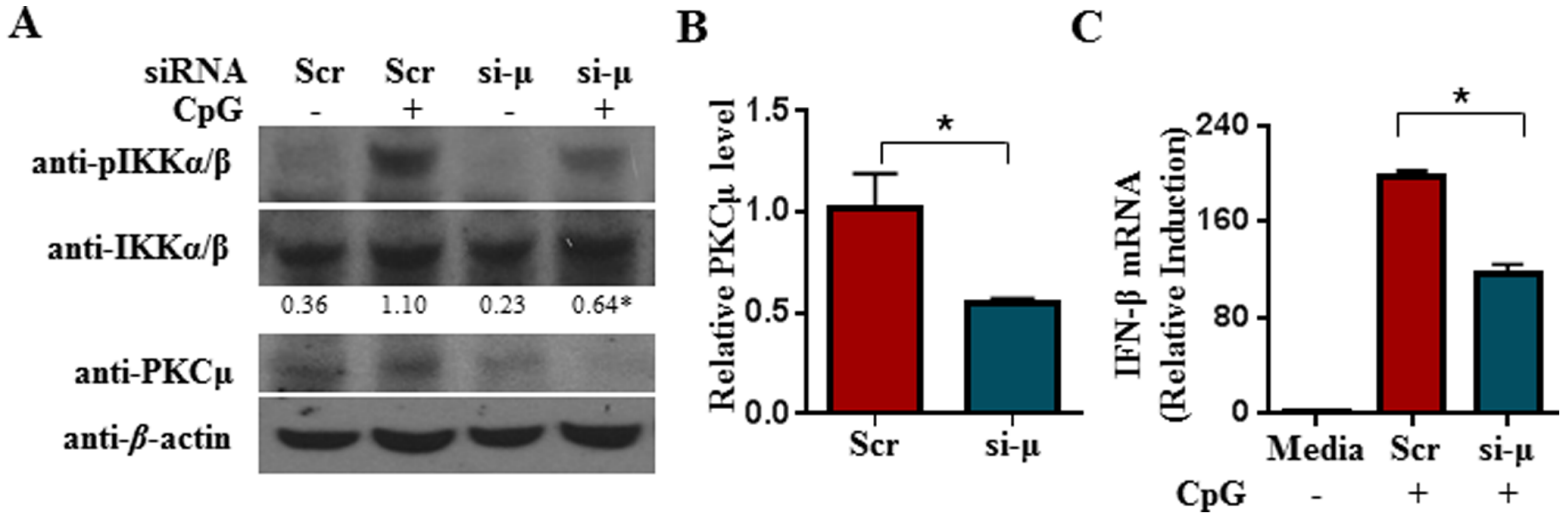
siRNA-mediated knockdown of PKCµ attenuated IKKα/β activation and IFN-β synthesis in CpG-stimulated cDC. PKCµ siRNA (si-µ) or scrambled siRNA (Scr) were transfected into wild type cDC for 24 h prior to the stimulation of cells with CpG for 1 h. (A) Expression of PKCµ and activation of IKKα were examined via western blot analyses using anti-PKCµ and anti-phospho-IKKα (Ser^180^)/IKKβ (Ser^181^) antibodies, respectively. The anti- IKKα/β and anti-β-actin blots served as loading control. Data shown are representative of 3 independent experiments. The intensity ratio of phospho-IKKα (Ser^180^)/IKKβ (Ser^181^) over IKKα/β is shown below the gels. *p<0.05 (Student's *t* test), compared to the second lane of CpG-treated cells transfected with scrambled siRNA. (B) PKCµ expression level normalized to β-actin in scramble or PKCµ siRNA transfected cDC. % PKCµ level was calculated with respect to scramble-transfected cells. (C) qRT-PCR analysis of IFN-β mRNA expression in CpG-stimulated cells with or without PKCµ-kockdown. Data were analyzed as in Fig. 2A. Numbers represent mean±SEM of three experiments. *p<0.05 (Student's *t* test).

To further confirm that PKCµ is critical for TLR7/9-induced IFN-β production, we knockdown PKCµ expression in primary cDC using siRNA technology. Purified cDC were harvested on day 6 of culture and transfected with either scrambled siRNA (Scr) or PKCµ-specific siRNA (si-µ). Cells were allowed to grow for an additional 24 h before stimulation with CpG. As shown in Fig. 6A & B, PKCµ expression could indeed be reduced by PKCµ-specific siRNA compared to scrambled siRNA treatment. As a control, we showed that the knockdown of PKCµ expression did not affect the expression of IKKα/β or β-actin. When PKCµ expression was successfully knocked-down, the activation of IKKα/β was attenuated in CpG-stimulated cDC. Consistent with the reduced activation of IKKα/β in the CpG-stimulated and si-PKCµ-transfected cDC, the synthesis of IFN-β mRNA was also defective (Fig. 6C).

Taken together, our data indicated that the reduction of PKCµ activity or expression perturbs the activation of IKKα/β and nuclear translocation of IRF-1 which in turn lead to impaired IFN-β production in TLR7/9-stimulated cDC.

## Discussion

The signaling pathway leading to IFN-β synthesis upon TLR7/9 stimulation in cDC has not been well characterized. Although it is well established that TLR3 and TLR4 could induce type 1 interferon production in cDC and macrophages through the activation of IRF-3 [48], [49], [50], most studies of TLR7/9-induced production of type 1 IFN had centered on the activation of the different IRFs in different cell types. For example, IRF-1 and IRF-7 have been shown to be important for IFN production in cDC and pDC, respectively [8], [11], [38]. In recent years, Kaisho and colleagues had expanded on these studies by showing that IKKα played an important role in TLR7/9 signaling of IFNα production in pDC [10] and IFN-β production in cDC [12]. In this paper, we further extended these analyses and sought to delineate the signaling partners that acts upstream of IKKα and IRF in the induction of IFN-β synthesis triggered by TLR7/9 engagement in cDC.

It was previously demonstrated that BTK played a critical role in MyD88/TLR-dependent proinflammatory cytokine production in various cell types [17], [18], [24]. However, a role for BTK in TLR7/9 signaling of IFN-β production in cDC has not been explored. Our current study shows that BTK is indispensable for IFN-β production in TLR7/9 activated cDC. More importantly, our data indicate that BTK acts upstream of IKKα/β and IRF-1 in the TLR7/9 signaling pathway leading to IFN-β production in cDC.

Other than BTK, our current work also establishes a role for PKCµ in the induction of IFN-β production by TLR7/9. Although studies have implicated PKCα and PKCµ in TLR-induced proinflammatory cytokine production [27], [45], [46] and PKCα in TLR3-induced IFN-β production via IRF-3 [29], it is not known if there is a role for any PKC in the induction of IFN synthesis through TLR7/9 and if so, which PKC isoform would be involved in this process. Our data demonstrate that PKCµ plays an important role in TLR7/9-signaling of IFN-β production in cDC and that it signals upstream of IKKα/β and IRF-1 activation. In addition, we identified BTK to be essential for PKCµ activation as its deficiency leads to defective PKCµ phosphorylation.

Although cDC possess other Tec family kinase and PKC isoforms, we found BTK and PKCµ to be non-redundant in TLR7/9 activation of IKKα/β and IRF-1 for the induction of IFN-β synthesis. Taken together, our study delineates a BTK-PKCµ-IKKα/β-IRF-1 signaling axis downstream of TLR7/9-signaling that is critical for the induction of IFN-β synthesis in cDC. Future work could be pursued to determine additional binding partners or signaling molecules in TLR7/9-induced IFN-β pathway in cDC.
